# Supplementary material for: Single-molecular insights into the breakpoint of cellulose nanofibers assembly during saccharification
Source: Nat Commun. 2023 Feb 25;14:1100. doi: 10.1038/s41467-023-36856-8 (PMC9968341; doi:10.1038/s41467-023-36856-8)
Supplement: Supplementary file 1 — Supplementary Information [file 41467_2023_36856_MOESM1_ESM.pdf]

Single-molecular insights into the breakpoint of cellulose nanofibers assembly during saccharification

Zhang and Hu *et al.*

Supplementary Table 1. Characteristic bands of the FT-IR spectra.

Reported wavenumber (cm^{-1})	Observed wavenumber (cm^{-1})	Functional group	Assignment
829	831	C─H breathing	H-lignin
898	898	C─H vibration	Cellulose
1051	1051	C─O─C ring skeletal vibration	Hemicelluloses
1163	1164	C─O─C asymmetric stretching	Cellulose
1247	1247	C─O─C stretching of aryl-alkyl ether	Lignin
1373	1371	C─H_2 scissoring	Cellulose
1430	1430	C─H_2 bending	Cellulose
1460	1460	C─H_3 asymmetric bending	Lignin
1515	1511	C=C stretching of the aromatic ring	Lignin
1590	1598	C=C stretching	Lignin
1603	1603	C=C stretching	Lignin
1735	1735	C=O stretching of acetyl or carboxylic acid	Hemicelluloses & Lignin
2900	2900	C─H stretching	Cellulose
3350	3378	O─H stretching	Cellulose

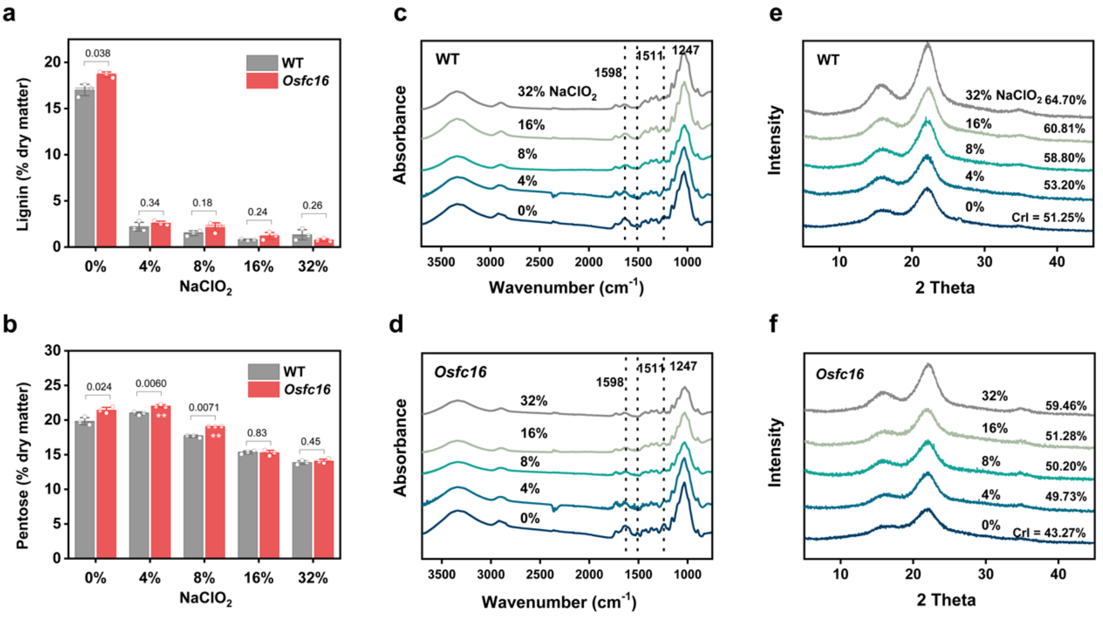

Supplementary Fig. 1. Wall polymers levels and properties in stem tissue residues of *Osfc16* mutant and WT after $NaClO_2$ treatments.

a Lignin content. **b** Pentoses content crudely as hemicellulose. Bars as means ± SD (n = 3 biologically independent samples). **c**, **d** Fourier transform infrared spectroscopic profiling. Information of chemical bonds for all major peaks listed in Supplementary Table 1. **e**, **f** XRD spectroscopic profiling. Significant differences between the WT and mutant were determined using two-tailed Student's *t*-test: $^{**}P < 0.01$, $^{*}P < 0.05$. Source data are provided as a Source Data file.

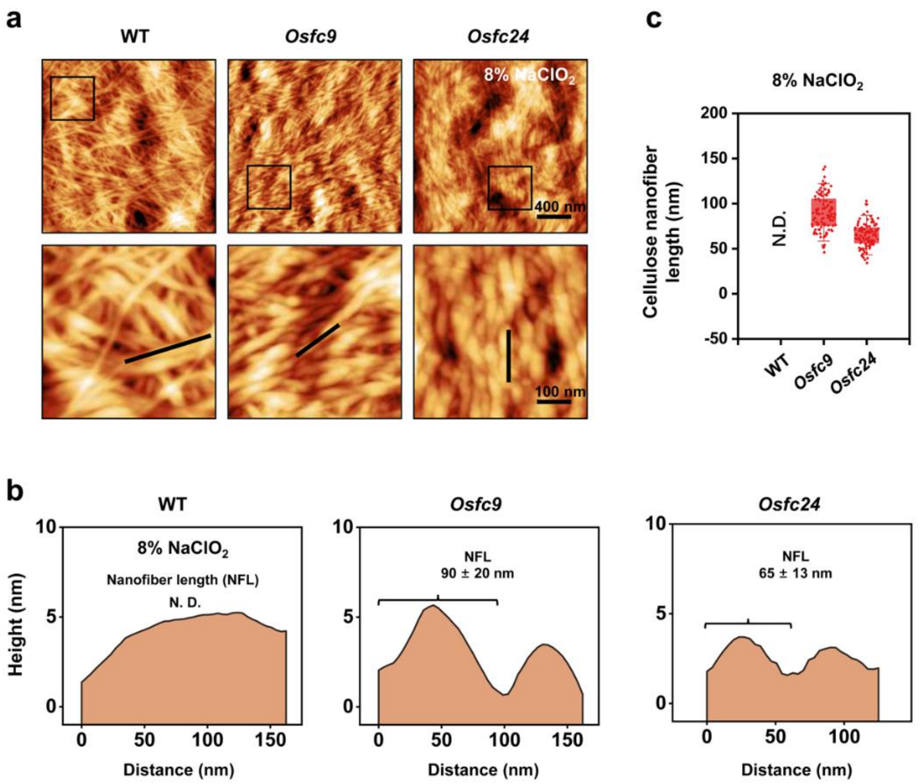

Supplementary Fig. 2. *In situ* measurements of cellulose defects in *Osfc9* and *Osfc24* mutants.

a Classic AFM topography in plant cell walls after 8% $NaClO_2$ treatment. **b** Cross profiles corresponding for black lines in (**a**) to illustrate alternate breakpoints for nanofibers lengths. **c** Average nanofibers lengths distribution in (**b**). NFL, nanofibers length, data as means ± SD (n = 100 nanofibers counted from 3 biologically independent samples). Data in (**c**) are displayed as box and whisker plots with individual data points. The error bars represent 95th and 5th percentiles. Center line, average; box limits, 25th and 75th percentiles. AFM experiments were repeated at least 3 times independently with similar results. Source data are provided as a Source Data file.

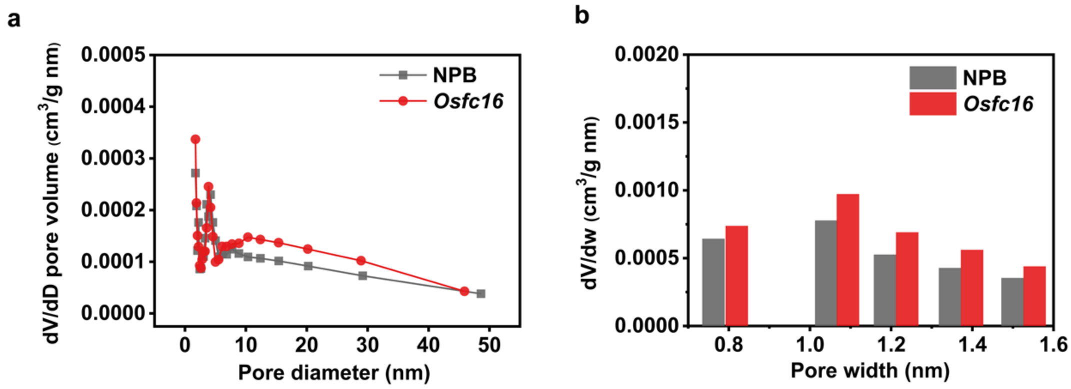

Supplementary Fig. 3. Raised nanopore volumes in *Osfc16* mutant.

a BJH adsorption micropore and **b** Horvath-Kawazoe nanopore distribution of crude cellulose substrates after 8% $NaClO_2$ extraction with mature straws. Source data are provided as a Source Data file.

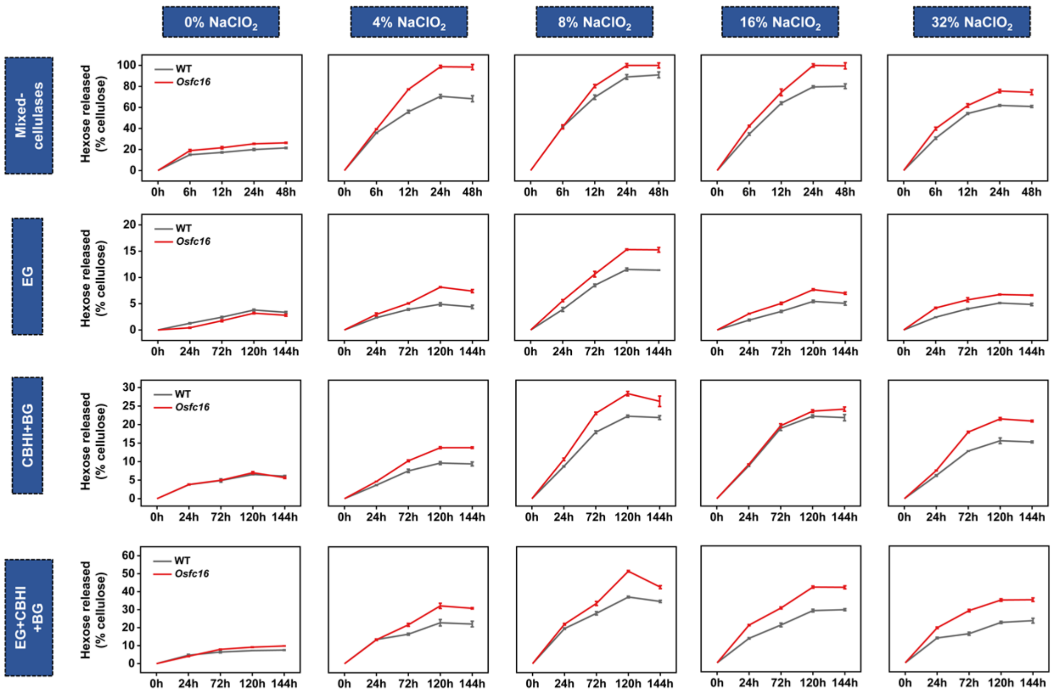

Supplementary Fig. 4. One-step enzymatic hydrolyses with stem powders samples of *Osfc16* mutant and WT after $NaClO_2$ treatments with time course. Bars as means ± SD (n = 3 biologically independent samples). Source data are provided as a Source Data file.
