# Supplementary material for: Single-molecular insights into the breakpoint of cellulose nanofibers assembly during saccharification
Source: Nat Commun. 2023 Feb 25;14:1100. doi: 10.1038/s41467-023-36856-8 (PMC9968341; doi:10.1038/s41467-023-36856-8)
Supplement: Supplementary file 3 — Reporting Summary [file 41467_2023_36856_MOESM3_ESM.pdf]

nature portfolio

Corresponding author(s): Liangcai Peng; Bingqian Xu; Haizhong Yu

Last updated by author(s): Feb 3, 2023

Reporting Summary

Nature Portfolio wishes to improve the reproducibility of the work that we publish. This form provides structure for consistency and transparency in reporting. For further information on Nature Portfolio policies, see our Editorial Policies and the Editorial Policy Checklist.

Statistics

For all statistical analyses, confirm that the following items are present in the figure legend, table legend, main text, or Methods section.

n/a	Confirmed	
☐	☒	The exact sample size (*n*) for each experimental group/condition, given as a discrete number and unit of measurement
☐	☒	A statement on whether measurements were taken from distinct samples or whether the same sample was measured repeatedly
☐	☒	The statistical test(s) used AND whether they are one- or two-sided<br>*Only common tests should be described solely by name; describe more complex techniques in the Methods section.*
☒	☐	A description of all covariates tested
☒	☐	A description of any assumptions or corrections, such as tests of normality and adjustment for multiple comparisons
☐	☒	A full description of the statistical parameters including central tendency (e.g. means) or other basic estimates (e.g. regression coefficient) AND variation (e.g. standard deviation) or associated estimates of uncertainty (e.g. confidence intervals)
☐	☒	For null hypothesis testing, the test statistic (e.g. *F*, *t*, *r*) with confidence intervals, effect sizes, degrees of freedom and *P* value noted<br>*Give P values as exact values whenever suitable.*
☒	☐	For Bayesian analysis, information on the choice of priors and Markov chain Monte Carlo settings
☒	☐	For hierarchical and complex designs, identification of the appropriate level for tests and full reporting of outcomes
☐	☒	Estimates of effect sizes (e.g. Cohen's *d*, Pearson's *r*), indicating how they were calculated

Our web collection on statistics for biologists contains articles on many of the points above.

Software and code

Policy information about availability of computer code

Data collection	PicoView (1.14) was used for data collection in the AFM.
Data analysis	PicoView (1.14) , Gwyddion (2.56) softwares were used to analyze and process AFM data. SPSS Statistics 26 software was used to conduct Spearman's correlation assay. The variation and regression analysis were completed using Origin 2018 software. Microsoft Excel 2016 was used for t-test.

For manuscripts utilizing custom algorithms or software that are central to the research but not yet described in published literature, software must be made available to editors and reviewers. We strongly encourage code deposition in a community repository (e.g. GitHub). See the Nature Portfolio guidelines for submitting code & software for further information.

Data

Policy information about availability of data

All manuscripts must include a data availability statement. This statement should provide the following information, where applicable:

- Accession codes, unique identifiers, or web links for publicly available datasets
- A description of any restrictions on data availability
- For clinical datasets or third party data, please ensure that the statement adheres to our policy

No databases or datasets generated or used in the study.

Human research participants

Policy information about studies involving human research participants and Sex and Gender in Research.

Reporting on sex and gender	N/A
Population characteristics	N/A
Recruitment	N/A
Ethics oversight	N/A

Note that full information on the approval of the study protocol must also be provided in the manuscript.

Field-specific reporting

Please select the one below that is the best fit for your research. If you are not sure, read the appropriate sections before making your selection.

☒ Life sciences ☐ Behavioural & social sciences ☐ Ecological, evolutionary & environmental sciences

For a reference copy of the document with all sections, see nature.com/documents/nr-reporting-summary-flat.pdf

Life sciences study design

All studies must disclose on these points even when the disclosure is negative.

Sample size	No statistical methods were used to determine sample size. Sample size is based on our previous publications. (Phys. Chem. Chem. Phys. 15, 6508-6515 (2013);Biotechnol. Biofuels 5, 58 (2012);Bioresource Technol. 369, 128437 (2022))
Data exclusions	No data were excluded from our analysis.
Replication	All attempts at replication were successful. For AFM observations, at least 3 independent trials (different plant cross sections) were conducted to make sure biological repeatability. For the measurement of nanofibers and nanocrystals, 100 data points were collected from 10 zoom-in areas of 3 biological independently samples were examined. For force distribution analysis, 300 curves were collected. For cell wall content, digestibility analysis and cellulose DP, three biological independent experiments were conducted. The XRD method was detected with SD at ±0.05–0.15 using five representative samples in triplicate to ensure method stability. For FT-IR and BET analyses, two biological replicates were included, and similar peak alteration were observed.
Randomization	The experiments were conducted to compare features between wild type and mutant. Wild type and mutant need to be judged by their phenotype first to make sure the sampling, thus randomization is not relevant to allocate WT vs mutant. But to allocate WT or mutant in their own experiment groups (different conditions or biological replication) are random.
Blinding	Wild type and mutant need to be judged by their phenotype first to make sure the sampling, thus blinding is not relevant to sampling in WT vs mutant. Experiments were not blind in AFM/BET/XRD/FT-IR because results are automatically given by equipments. Cell wall composition, digestibility, cellulose DP analysis were done by different persons, and they were blinded when allocating samples or collecting data.

Reporting for specific materials, systems and methods

We require information from authors about some types of materials, experimental systems and methods used in many studies. Here, indicate whether each material, system or method listed is relevant to your study. If you are not sure if a list item applies to your research, read the appropriate section before selecting a response.

Materials & experimental systems

n/a	Involved in the study	
☒	☐	Antibodies
☒	☐	Eukaryotic cell lines
☒	☐	Palaeontology and archaeology
☒	☐	Animals and other organisms
☒	☐	Clinical data
☒	☐	Dual use research of concern

Methods

n/a	Involved in the study	
☒	☐	ChIP-seq
☒	☐	Flow cytometry
☒	☐	MRI-based neuroimaging
